# Tendência temporal de equipes de saúde bucal da Estratégia Saúde da Família nos municípios brasileiros de 2001 a 2021

**DOI:** 10.1590/0102-311XPT169424

**Published:** 2025-06-20

**Authors:** Lucas Xavier Bezerra de Menezes, Gabriel Trevizan Corrêa, Edson Hilan Gomes de Lucena, Roger Keller Celeste, Yuri Wanderley Cavalcanti

**Affiliations:** 1 Universidade Federal da Paraíba, João Pessoa, Brasil.; 2 Faculdade de Odontologia, Universidade Federal do Rio Grande do Sul, Porto Alegre, Brasil.; 3 Aging Research Center, Karolinska Institut, Solna, Sweden.

**Keywords:** Estratégias de Saúde Nacionais, Atenção Primária à Saúde, Saúde Bucal, National Health Strategies, Primary Health Care, Oral Health, Estrategias de Salud Nacionales, Atención Primaria de Salud, Salud Bucal

## Abstract

O objetivo deste estudo foi descrever e analisar a tendência das taxas de equipes de saúde bucal da Estratégia Saúde da Família (ESF) no período de 2001 a 2021. É um estudo ecológico em nível municipal com os 5560 municípios existentes no ano de 2002. A fonte de dados foi do portal governamental e-Gestor Atenção Básica que permite acesso a vários Sistemas de Informação em Saúde. A variável dependente foi a taxa de equipes de saúde bucal por 100 mil habitantes/ano. As variáveis independentes foram: taxa de equipes de saúde da família, macrorregião brasileira, porte populacional, PIB *per capita* e a implantação da Política Nacional de Saúde Bucal, em 2004, e da *Emenda Constitucional nº 95*, em 2016. Utilizou-se modelo de regressão linear generalizado com o método Prais-Winsten. Verificou-se um crescimento constante de equipes ao longo do período, porém, com gradual desaceleração. A taxa passou de 1,9 em 2001 para 29 equipes de saúde bucal por 100 mil habitantes em 2021, um crescimento total de 27,1%. Após 2004, houve um acréscimo de 1,8 equipe por 100 mil habitantes (IC95%: 1,7; 2,0). Após 2016, ocorreu redução média de 0,5 (IC95%: -0,6; -0,3) em relação ao crescimento anual. Municípios com menor porte populacional, menor PIB *per capita* e localizados na Região Nordeste tiveram taxas de aumento acima da tendência média (32,9, 16,2 e 33,6, respectivamente). Observa-se que as equipes de saúde bucal da ESF têm se expandido em regiões com maior necessidade social de serviços. Mais estudos são necessários a fim de investigar outros fatores que influem sobre a variação na série histórica da saúde bucal na saúde da família.

## Abstract (en)

This study aimed to describe and analyze the trend in the rates of Family Health Strategy (FHS) oral health teams from 2001 to 2021. The 5,560 municipalities existing in Brazil in 2002 were included in this ecological study. The data were extracted from the government portal *e-Gestor Atenção Básica* (e-Manager Primary Care), which offers access to various health information systems. The rate of oral health teams per 100,000 inhabitants/year was considered as the dependent variable. Rate of family health teams, Brazilian macroregion, population size, GDP per capita, and the implementation of the Brazilian National Oral Health Policy in 2004 and *Constitutional Amendment n. 95* in 2016 were chosen as independent variables. A generalized linear regression model was used with the Prais-Winsten method. Teams steadily grew throughout the period, but such increase gradually decelerated over time. Rates went from 1.9 in 2001 to 29 oral health teams per 100,000 inhabitants in 2021, a total growth of 27.1. After 2004, teams increased by 1.8 teams per 100,000 inhabitants (95%CI: 1.7; 2.0). After 2016, annual growth showed an average reduction of 0.5 (95%CI: -0.6; -0.3). Municipalities with smaller populations, lower GDP per capita, and those in the Brazilian Northeast showed increase rates above the average trend (32.9, 16.2, and 33.6, respectively). FHS oral health teams have grown in regions with greater social need for services. Further studies are needed to investigate other factors that influence the variation in the historical series of oral family health.

## Resumen

El objetivo de este estudio fue describir y analizar la tendencia de las tasas de equipos de salud bucal de la Estrategia de Salud Familiar (ESF) en el período de 2001 a 2021. Se trata de un estudio ecológico, a nivel municipal, con 5.560 municipios en 2002. La fuente de datos fue la página gubernamental *e-Gestor Atenção Básica*, que permite el acceso a varios Sistemas de Información de Salud. La variable dependiente fue la tasa de equipos de salud bucal por cada 100.000 habitantes/año. Las variables independientes fueron: tasa de equipos de salud familiar, macrorregión brasileña, tamaño de la población, PIB per cápita e implementación de la Política Nacional de Salud Bucal en 2004 y la *Enmienda Constitucional n. 95* de 2016. Se utilizó un modelo de regresión lineal generalizado con el método Prais-Winsten. Sin embargo, hubo un crecimiento constante de los equipos durante el período, con una desaceleración gradual. La tasa pasó de 1,9 en 2001 a 29 equipos de salud bucal por cada 100.000 habitantes en 2021, un crecimiento total de 27,1. Tras 2004, hubo un aumento de 1,8 equipos por cada 100.000 habitantes (IC95%: 1,7; 2,0). Tras 2016, hubo una reducción promedio de 0,5 (IC95%: -0,6; -0,3) en relación con el crecimiento anual. Los municipios con menor tamaño poblacional, menor PIB per cápita y ubicados en la región Nordeste de Brasil tuvieron tasas de incremento por encima de la tendencia promedio (32,9, 16,2 y 33,6, respectivamente). Se observa que los equipos de salud bucal de la ESF se han expandido en regiones con mayor necesidad social de servicios. Se necesitan más estudios para investigar otros factores que influyen en la variación en la serie histórica de salud bucal en la salud familiar.

## Introdução

A Estratégia Saúde da Família (ESF) surge com o objetivo de reorientar a atenção em saúde no Brasil, sendo reconhecida como uma estratégia de expansão e qualificação da atenção primária ^1^. No final do ano 2000, as equipes de saúde bucal (EqSB) foram introduzidas à ESF ^2^, na proporção de uma EqSB para cada duas equipes de saúde da família (EqSF), e, em 2003, na proporção de um para um ^3^. Contudo, apenas em 2004 foi publicada a Política Nacional de Saúde Bucal - Brasil Sorridente (PNSB), com o objetivo de reorganizar a atenção em saúde bucal em todos os níveis de atenção. As diretrizes apresentadas na PNSB reorientam as concepções e práticas no âmbito da saúde bucal, rompendo com a prática odontológica tradicional ^4^ ^,^ ^5^.

A partir da PNSB, foram observados grandes avanços na ampliação e qualificação da atenção em saúde bucal no Brasil. Como exemplos, temos o aumento da força de trabalho, os reajustes nos repasses financeiros e mais recursos destinados à área, um maior investimento na capacidade instalada dos serviços e inclusão de novos procedimentos e tecnologias na atenção primária ^6^. Já com relação ao quantitativo de EqSB e ao acesso e cobertura da população, houve um crescimento superior a 470% no número das equipes e uma ampliação de mais de 378% na cobertura populacional das mesmas no Brasil, entre os anos de 2002 e 2016 ^7^.

Embora sejam observados os avanços significativos, muitos desafios ainda podem ser encontrados na atenção em saúde bucal dos municípios brasileiros. As condições de trabalho, a ampliação e qualificação da assistência, o monitoramento e avaliação das ações e o trabalho intersetorial são algumas das dificuldades ainda enfrentadas pelas EqSB no processo de trabalho ^8^. Do ponto de vista socioeconômico, a instabilidade política e econômica vivenciada nos últimos anos também se tornou um desafio para a saúde bucal. Neste sentido, destaca-se a aprovação da *Emenda Constitucional nº 95* de 2016 (EC95) ^9^, que limitou o teto de gastos para saúde, educação e previdência social ^10^ ^,^ ^11^ ^,^ ^12^.

Dado o crescimento na cobertura de EqSB no Brasil, é improvável que ele tenha ocorrido de forma igualitária em todo o país. Sob essa perspectiva, monitorar e analisar indicadores de acesso, uso e cobertura dos serviços odontológicos, bem como informações sobre as EqSB, torna-se fundamental para nortear a elaboração e revisão da política da área ^13^ ^,^ ^14^. Acrescenta-se a isso o fato da PNSB completar 20 anos em 2024, sendo relevante avaliar sua evolução ao longo destes anos.

Diante do exposto, o presente estudo busca analisar a tendência das taxas de EqSB da ESF no período de 2001 a 2021 nos municípios brasileiros, segundo variáveis tempo-dependente e contextuais. Pretende-se, com isto, avaliar o efeito de políticas nacionais na série e explorar tendências nas desigualdades de cobertura das EqSB.

## Metodologia

### Desenho do estudo

Realizou-se um estudo longitudinal de série temporal, ecológico e observacional, descritivo e analítico, cujas unidades de observação são os municípios brasileiros. Analisou-se anualmente a quantidade de EqSB da ESF, credenciadas pelo Ministério da Saúde, no período de 2001 a 2021, sendo esta a variável primária. Variáveis secundárias foram: PNSB (Brasil Sorridente) e EC95; macrorregião, porte populacional e produto interno bruto (PIB) *per capita* dos municípios.

### Coleta de dados e constituição da amostra

A quantidade absoluta e percentual de cobertura de EqSB e EqSF por município/mês foi obtida nos Painéis de Indicadores da Atenção Primária à Saúde (https://sisaps.saude.gov.br/painelsaps), dispostos no portal governamental e-Gestor Atenção Básica, do Ministério da Saúde (https://egestorab.saude.gov.br/). Estes dados, disponíveis mensalmente, foram agrupados anualmente, uma vez que não foram verificadas variações significativas ao longo do ano. A macrorregião, o porte populacional e o PIB *per capita* foram obtidos do Instituto Brasileiro de Geografia e Estatística (IBGE). Os dois bancos de dados utilizados são de acesso público e a coleta de dados foi realizada do mês de fevereiro a junho de 2023. Foram considerados elegíveis para o estudo todos os municípios brasileiros existentes em 2001. Todos os demais, criados de 2002 em diante, não foram incluídos.

### Constituição do modelo

Foram utilizadas as variáveis tempo-dependentes: (1) antes (≤ 2004) e depois (> 2004) da implantação do Brasil Sorridente; e (2) antes (≤ 2017) e depois (> 2017) da EC95 (Regime Fiscal que instituiu o teto de gastos na saúde). As variáveis municipais contextuais foram: (1) taxa de EqSF por 100 mil habitantes/ano (0-15; 16-30; 31-45 e maior que 45); (2) macrorregião do país (Norte; Nordeste; Sudeste; Sul e Centro-oeste); (3) porte populacional dos municípios (até 10 mil habitantes; de 10.001 a 20 mil habitantes; 20.001 a 100 mil habitantes e > 100 mil habitantes); e (4) o PIB *per capita*, em 2010 (até BRL 3.500,00; BRL 3.501,00 a BRL 7.000,00; BRL 7.001,00 a BRL 10.500,00; e > BRL 10.500,00). Para porte populacional e PIB *per capita* foram utilizados valores fixos referentes ao ano de 2010. Isto foi realizado por haver uma correlação linear r > 0,95 dentre medidas de diferentes anos ao longo do período analisado. A variável dependente considerada no modelo foi a taxa municipal de EqSB por 100 mil habitantes/ano, sendo essa taxa correspondente ao número total de EqSB dividido pela população de cada ano correspondente, sendo assim ajustado de acordo com as variações populacionais dos municípios.

### Análise estatística

As variáveis quantitativas foram analisadas descritivamente por meio de média e desvio padrão. Para variáveis categóricas foram obtidas as distribuições absoluta e relativa. Os resultados foram apresentados com seus respectivos intervalo de 95% de confiança (IC95%). Foram apresentadas análises comparativas das taxas municipais anuais de EqSB para cada covariável, nos anos de 2001 e 2021, usando o teste Kruskal-Wallis para dados não normalmente distribuídos. Em seguida, realizou-se modelo de regressão linear generalizado utilizando o método Prais-Winsten auto-regressivo (AR1), para as taxas municipais de EqSB por 100 mil habitantes/ano. Este método é indicado para corrigir a autocorrelação serial em séries temporais ^15^ ^,^ ^16^. Interações entre variáveis fixas e ano foram ajustadas para testar crescimento diferencial. Todas as análises foram realizadas no software Stata 16.2 (https://www.stata.com).

## Resultados

Em 2001, havia 5.560 municípios no Brasil e em 2021, 5.570. Todos os municípios existentes no ano-base foram elegíveis para o estudo, não sendo incluídos, portanto, todos os 10 criados de 2002 até o ano final. Aqueles com maiores taxas de EqSF (> 45 por 100 mil habitantes/ano) e os que possuíam menos de 10 mil habitantes apresentaram maiores taxas de EqSB nos anos base e final. Quanto ao PIB, em 2001, os municípios com maiores taxas de EqSB se concentravam no grupo de maior atividade econômica *per capita*. Em 2021 houve uma inversão, as maiores taxas foram observadas nos municípios de menor PIB. Quanto à macrorregião brasileira, os municípios do Centro-oeste apresentaram as maiores taxas de EqSB em 2001 e, em 2021, os do Nordeste. A variação das taxas de EqSB entre as categorias foram estatisticamente significativas para todas as variáveis contextuais nos dois anos (p < 0,01), exceto para porte municipal em 2001 (Tabela 1).

**Tabela 1:** Taxas média municipais e desvios padrão (DP) de equipes de saúde bucal (EqSB) por covariáveis nos anos de 2001 e 2021 em 5.560 municípios brasileiros.

	Total		Taxa de EqSB por 100.000 habitantes/ano (2001)			Taxa de EqSB por 100.000 habitantes/ano (2021)		
	n	%	Média municipal	DP	Valor de p	Média municipal	DP	Valor de p
Total	5.560	100,0	1,9	6,4		29,0	17,0	
Taxa de EqSF por 100.000 habitantes/ano								
0-15			0,2	1,3	< 0,01	6,3	7,7	< 0,01
15-30			3,1	6,2		16,6	9,7	
30-45			7,1	12,0		30,6	12,0	
> 45			12,3	21,0		45,0	17,0	
Macrorregião								
Centro-oeste	463	8,3	6,4	12,0	< 0,01	31,7	15,0	< 0,01
Nordeste	1.792	32,0	3,1	7,2		36,7	13,0	
Norte	449	8,1	0,3	1,5		27,0	18,0	
Sudeste	1.668	30,0	0,5	3,7		23,9	18,0	
Sul	1.188	21,0	1,1	5,2		24,2	18,0	
Porte municipal em 2010								
Até 10.000	2.508	45,0	3,1	9,0	0,10	36,0	18,0	< 0,01
10.001-20.000	1.401	25,0	1,2	3,0		28,5	14,0	
20.001-100.000	1.368	25,0	0,9	2,2		20,7	13,0	
> 100.000	283	5,1	0,4	1,0		9,6	7,3	
PIB *per capita* em 2010								
< BRL 3.500	1.163	21,0	2,7	6,5	< 0,01	37,2	14,0	< 0,01
BRL 3.500-7.000	1.523	27,0	2,1	7,0		33,2	16,0	
BRL 7.000-10.500	1.250	23,0	1,8	7,0		25,6	18,0	
> BRL 10.500	1.624	29,0	1,2	5,1		21,8	16,0	

EqSF: equipes de saúde da família; PIB: produto interno bruto.

**Figura 1:**
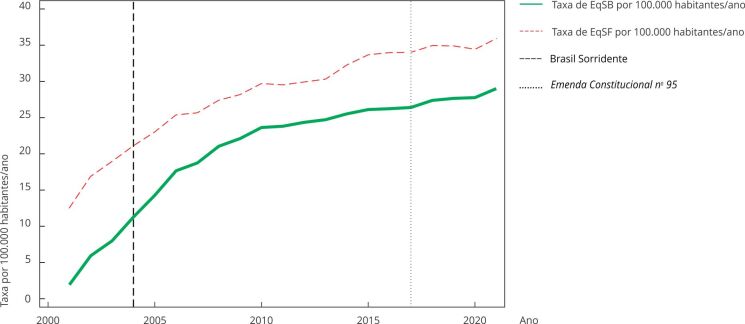
Taxa média municipal de equipes de saúde bucal (EqSB) por 100 mil habitantes no Brasil com o destaque da implantação do Brasil Sorridente (em 2004) e *Emenda Constitucional nº 95/*2016, de 2001 a 2021.

A taxa média municipal de EqSB por 100 mil habitantes/ano foi igual a 1,9 em 2001, passando para 29,0 em 2021, um incremento de 27,1 unidades, ou de 15 a quantidade do ano-base. A taxa média municipal de EqSF, por sua vez, passou de 12,5 para 35,9 unidades por 100 mil habitantes/ano nos 21 anos estudados. São 23,4 EqSF a mais, que corresponde a um aumento de apenas 2,8 vezes o valor de 2001. Quanto à cobertura percentual das equipes, a de saúde bucal era de 6,1% em 2001, passando para 76,2% da população brasileira em 2021 (crescimento de 70,1 pontos percentuais, ou de 12,5 vezes o percentual do ano-base). Já a cobertura de EqSF, que era de 39,1%, alcançou 90,5% dos brasileiros em 2021 (incremento de 51,4 pontos percentuais, ou de 2,3 vezes o percentual de 2001) (Tabela 2 e Figura 1).

**Tabela 2:** Taxas médias municipais por 100 mil habitantes/ano e desvios padrão (DP) de equipes de saúde bucal (EqSB) e equipes de saúde da família (EqSF) em 5.560 municípios brasileiros, de 2001 a 2021.

Ano	Taxa de EqSB por 100.000 habitantes/ano		Taxa de EqSF por 100.000 habitantes/ano		% de cobertura de EqSB		% de cobertura de EqSF	
	Média municipal	DP	Média municipal	DP	Média municipal	DP	Média municipal	DP
2001	1,9	6,4	12,5	13,8	6,1	18,5	39,1	39,0
2002	5,9	10,8	16,9	14,7	18,5	30,0	51,6	39,9
2003	8,0	12,0	18,9	15,0	24,8	32,9	57,0	39,2
2004	11,3	14,0	21,1	14,8	34,4	37,8	62,9	37,2
2005	14,3	15,0	23,0	14,5	42,8	39,5	67,8	35,4
2006	17,7	15,6	25,4	14,2	52,0	39,8	73,5	33,3
2007	18,8	15,3	25,7	14,0	55,5	39,0	74,5	32,4
2008	21,0	15,6	27,4	13,9	61,3	38,5	78,0	31,0
2009	22,1	15,5	28,2	13,7	64,1	37,8	79,7	30,0
2010	23,6	15,8	29,7	14,0	67,1	37,0	81,8	29,1
2011	23,8	15,4	29,5	13,7	68,1	36,4	82,0	28,6
2012	24,4	15,4	29,9	13,6	69,4	35,8	82,8	27,7
2013	24,7	15,3	30,3	13,4	70,2	35,4	83,6	27,0
2014	25,5	15,7	32,2	13,5	71,3	35,0	86,4	24,4
2015	26,1	16,0	33,7	13,4	72,0	34,6	88,4	22,0
2016	26,2	16,1	33,9	13,3	72,1	34,5	88,9	21,4
2017	26,4	16,2	34,0	13,5	72,2	34,6	88,8	21,6
2018	27,4	16,5	34,9	13,6	73,7	34,2	89,9	20,3
2019	27,7	16,7	34,9	13,8	74,0	34,2	89,6	20,7
2020	27,8	16,9	34,4	14,6	74,0	34,2	88,3	22,3
2021	29,0	17,2	35,9	14,3	76,2	33,2	90,5	19,9

Nota: médias não ponderadas pelo tamanho populacional municipal.

EqSF: equipes de saúde da família.

O modelo 3 ajustado da regressão linear Prais-Winsten, dos três estruturados, é o que melhor explica a variabilidade da taxa de EqSB pelas covariáveis (11,2%). Verificou-se uma tendência de aumento contínuo da taxa de EqSB por 100 mil habitantes/ano de, em média, 1,3 unidade (mudança de ângulo). Ademais, o Brasil Sorridente esteve associado a um aumento médio de quase 2 unidades dessa taxa no ano seguinte à sua implantação (mudança de nível), além da média anual anterior. Por outro lado, a EC95 esteve associada a um efeito contrário: a de redução de meia equipe na velocidade de aumento da taxa média anual de EqSB, no ano subsequente à aprovação da Emenda (Tabela 3).

**Tabela 3:** Coeficientes de regressão linear Prais-Winsten autorregressivas (AR1) para taxas municipais anuais de equipes de saúde bucal (EqSB) por covariáveis.

Covariáveis	Modelo 1		Modelo 2		Modelo 3	
	Taxa de EqSB por 100.000 habitantes/ano	IC95%	Taxa de EqSB por 100.000 habitantes/ano	IC95%	Taxa de EqSB por 100.000 habitantes/ano	IC95%
Intercepto	3,6	3,1; 4,1	10,3	9,2; 11,4	6,4	4,9; 8,0
Brasil Sorridente						
≤ 2004		Referência		Referência		Referência	
> 2004		1,8	1,6; 2,0	1,8	1,7; 2,0	1,8	1,7; 2,0
*Emenda Constitucional nº 95*/2016						
≤ 2017		Referência		Referência		Referência	
> 2017		-0,4	-0,5; -0,2	-0,5	-0,6; -0,3	-0,5	-0,6; -0,3
Tendência anual	1,3	1,2; 1,3	0,9	0,9; 1,0	1,3	1,2; 1,4
Taxa de EqSF por 100.000 habitantes/ano						
0-15			Referência		Referência	
15-30			4,0	3,8; 4,2	4,1	3,9; 4,3
30-45			9,2	9,0; 9,4	9,2	9,0; 9,4
> 45			14,1	13,8; 14,4	13,8	13,5; 14,0
Macrorregião						
Centro-oeste			Referência		Referência	
Nordeste			0,1	-0,9; 1,1	-2,2	-3,6; -0,8
Norte			-4,4	-5,5; -3,3	-4,7	-6,3; -3,1
Sudeste			-6,6	-7,5; -5,7	-6,0	-7,2; -4,8
Sul			-5,9	-6,8; -5,0	-4,4	-5,6; -3,1
Porte municipal em 2010						
Até 10.000			Referência		Referência	
10.001-20.000			-5,8	-6,3; -5,2	-2,3	-3,1; -1,5
20.001-100.000			-8,2	-8,8; -7,7	-2,4	-3,1; -1,6
> 100.000			-10,8	-11,9; -9,8	-2,3	-3,8; -0,9
PIB *per capita* em 2010						
< BRL 3.500,00			Referência		Referência	
BRL 3.501,00-7.000,00			0,2	-0,5; 0,9	0,6	-0,4;1,6
BRL 7.001,00-10.500,00			-1,2	-2,1; -0,3	1,0	-0,3; 2,2
> BRL 10.500,00			-1,9	-2,8; -1,0	0,6	-0,6; 1,9
Termos de interação						
Nordeste * ano					0,2	0,1; 0,3
Norte * ano					0,0	-0,1; 0,1
Sudeste * ano					-0,1	-0,1; 0,0
Sul * ano					-0,2	-0,2; -0,1
10.000-20.000 * ano					-0,4	-0,4; -0,3
20.000-100.000 * ano					-0,6	-0,7; -0,5
> 100.000 * ano					-0,9	-1,0; -0,8
BRL 3.500-7.000 * ano					0,0	-0,1; 0,0
BRL 7.000-10.500 * ano					-0,2	-0,3; -0,1
> BRL 10.500 * ano					-0,3	-0,3; -0,2
Indicadores de ajuste do modelo						
BIC	744.310,4		733.084,3		732.148,5	
Pseudo-R^2^	0,0%		10,5%		11,2%	

BIC: *Bayesian information criterion*; EqSF: equipes de saúde da família; IC95%: intervalo de 95% de confiança; PIB: produto interno bruto.

Ao analisar as interações das covariáveis por ano, verificou-se que os municípios com > 45 EqSF/100 mil habitantes/ano apresentaram maior crescimento na taxa de EqSB. Em relação às macrorregiões do país, a Região Sul, ao longo do tempo, cresceu menos do que a Região Centro-oeste. A Região Nordeste foi a única que cresceu mais do que o Centro-oeste. Os municípios com mais de 100 mil habitantes e com maior PIB *per capita* cresceram menos ao longo dos anos (Tabela 3).

## Discussão

Os resultados mostraram que a taxa de EqSB cresceu anualmente no período 2001-2021, sob influência da implantação da PNSB e do teto de gastos da saúde em 2017. Além disso, observou-se que o crescimento da taxa de EqSB foi mais acentuado nos municípios com menor porte populacional, menor PIB *per capita* e naqueles localizados na Região Nordeste. Tais diferenças na tendência de crescimento de EqSB apontam para a direção da equidade no acesso aos serviços, uma vez que tais características dos municípios correspondem ao baixo desenvolvimento econômico e social, associados a maior demanda. De fato, pouco mais de 1/3 (34%) dos municípios brasileiros (n = 1.915) não possuem serviços privados de saúde, dependendo exclusivamente do Sistema Único de Saúde (SUS); 92% destes municípios têm menos de 20 mil habitantes.

O presente estudo observou um crescimento de aproximadamente 1.350% no quantitativo de EqSB por 100 mil habitantes no período de 2001 (1,9) a 2021 (29), após a avaliação da série temporal de força de trabalho em saúde bucal. Tivemos conhecimento de apenas um estudo com o mesmo tipo de análise: um trabalho analisou a tendência de cirurgiões-dentistas no Brasil, no período de 2007 a 2014, e encontrou aumento constante da quantidade destes profissionais ^16^. No setor público, entretanto, este aumento foi menor em comparação com o setor privado, tanto em relação aos clínicos gerais (0,5% de aumento) quanto aos especialistas (11,6%). Neste estudo, a área da ESF foi considerada uma dentre as 22 especialidades descritas para cirurgiões-dentistas no Cadastro Nacional de Estabelecimentos de Saúde (CNES) ^17^. 

Apesar de o nosso estudo apresentar aumento médio na taxa municipal, houve municípios que apresentaram redução no número de EqSB. Em outro estudo, que utilizou a análise de regressão de Cox, foi constatado que 6,7% dos municípios brasileiros reduziram a quantidade de EqSB ^6^. Em convergência com achados da nossa investigação, os municípios com maior porte populacional apresentaram maior redução na quantidade de EqSB e maior chance de reduzir a cobertura dessas. Por outro lado, o mesmo estudo mostrou que a Região Nordeste foi a segunda que mais reduziu o quantitativo de EqSB, assim como apresentou maior chance de redução no número de equipes, comparada à Região Norte ^6^.

O aumento da taxa média populacional de EqSB demonstra preocupação dos governos para com o cuidado em saúde bucal da população brasileira. Um relatório recente da Organização Mundial da Saúde (OMS) ^18^ apresenta resultados preocupantes em nível global: as doenças orais atingem cerca de metade da população mundial. Cárie dentária, doença periodontal severa, edentulismo e câncer de lábio são as mais prevalentes. Tendo em vista que um estado saudável da cavidade bucal e suas estruturas associadas é de suma importância para o pleno desenvolvimento das atividades do ser humano, sendo fundamental tanto para sua capacidade de se alimentar, comunicar e interagir, quanto prevenir a instalação e o desenvolvimento de diversas doenças ^16^. Embasando a necessidade de oferta crescente de força de trabalho em saúde bucal, conforme tendência encontrada nos resultados do presente estudo.

Acreditamos na plausibilidade de que o maior aumento médio nas taxas de EqSB ocorrido nos municípios menos populosos, mais pobres e localizados na Região Nordeste, encontrado em nosso estudo, não tenha sido ao acaso. O Brasil Sorridente propôs-se a expandir as EqSB por todo o território nacional a fim de fortalecer a atenção primária e ampliar o acesso da população à saúde bucal. Para isto, e para seus demais objetivos, nos primeiros 10 anos da política foram gastos USD 2,6 bilhões ^19^. De fato, de 2007 a 2015 houve um aumento de 118% nos municípios com EqSB implantadas. Neste último ano, 90% dos municípios brasileiros apresentavam EqSB. A cobertura populacional, por sua vez, aumentou 10,46% no período ^20^ ^,^ ^21^. Todo este incentivo e aumento não ocorreu somente nos centros urbanos, mas sobretudo, nas áreas com maiores necessidades sociais e escassez de oferta de serviços de saúde bucal. Além disso, expressiva maioria dos profissionais que atuam nas EqSB são concursados, o que pode ser considerado um fator motivador fundamental (embora não o único) para atuação em regiões onde antes havia vazios sanitários ^22^. Sugere-se, neste sentido, que os mecanismos de contratação de recursos humanos em saúde entrem na agenda da PNSB, assim como atuação do Estado na busca de transparência e corresponsabilidade dos municípios ^20^ ^,^ ^21^.

O delineamento longitudinal e a utilização de metodologia de série temporal destacam-se como pontos fortes deste estudo, em que se analisaram 21 pontos anuais. O fato de não nos limitarmos à análise bruta, mas ajustarmos o modelo por covariáveis tempo-dependentes importantes, como fatores estruturais, demográficos e socioeconômicos dos municípios, também se traduz em potencialidade. Além disso, nosso principal desfecho não correspondeu à tradicional cobertura percentual, embora análises de sensibilidade com o percentual tenham gerado as mesmas conclusões. O uso de percentual de cobertura, apesar de ser uma medida útil, é baseada em um critério arbitrário fixado em legislação ^1^ - ignora que a população municipal é dinâmica e estabelece um teto (100%). O uso de taxas por habitantes permite maior discriminação para avaliação quantitativa da oferta de serviços de saúde em nível populacional. Ainda, este é generalizável para todo o Brasil, uma vez que o resultado foi calculado para todos os municípios, eliminando a possibilidade de vieses de seleção, assim como foi ajustado para cada macrorregião. Por fim, nossas análises podem ser facilmente reproduzidas, pois foram utilizados dados secundários oficiais de acesso público. Neste sentido, em relação à alimentação dos dados de recursos humanos por parte da gestão pública, espera-se que haja ausência ou o mínimo de vieses de sub e de super notificação. De fato, não foram encontrados *outliers* nos dados coletados.

Quanto às limitações do estudo, o aumento de 1,8 EqSB em 2005, e a redução de 0,5 EqSB por 100 mil habitantes em 2017 não são, necessariamente, consequências exclusivas da implantação da PNSB e da aprovação da EC95, respectivamente. A respeito disso, o ajuste do modelo de regressão é estruturado de forma a ter o maior poder de explicação da variação do desfecho com a menor quantidade possível de variáveis. Entretanto, há de se reconhecer que a variação de taxa de EqSB encontrada pode estar associada a outros fatores além das seis covariáveis elencadas. Ainda, as relações estatisticamente significativas encontradas são de associação, o que presume, mas não confirma a causalidade. Estudos ecológicos futuros poderão utilizar, por exemplo, os índices de desenvolvimento humano (IDH) e o de Gini como variáveis explicativas. Este tem a vantagem de medir a desigualdade econômica da região estudada em contraposição à produção total de riqueza. Estudos avaliaram a relação entre processo e resultado em saúde bucal e as variáveis contextuais mencionadas, além da cobertura de EqSB: altas taxas de extração dentária estiveram associadas a municípios com baixo IDH e àqueles com alta cobertura de EqSB ao longo do tempo ^23^; em relação à dor dentária, tais exposições contextuais não estiveram associadas à ocorrência do sintoma ^24^ ^,^ ^25^. Estudos deste tipo, mas com cobertura de EqSB como variável dependente, não foram encontrados.

Outra limitação que gostaríamos de pontuar é o fato de que as análises deste estudo não contemplam as distintas modalidades de EqSB. Tal inclusão seria interessante para descrever o cenário específico de variação de equipes segundo modalidade, tendo em vista a importância dos profissionais auxiliar e técnico em saúde bucal para a maior resolutividade e transformação do modelo de atenção ^26^. A inclusão desses profissionais foi por muitos anos um tabu na história da saúde bucal brasileira ^27^, sendo em parte superada com a inclusão das EqSB na Saúde da Família. A discrepância acentuada nas quantidades de equipes segundo modalidade (praticamente 1 EqSB II para cada 10 EqSB I) ^21^, entretanto, demonstra que ainda há enorme resistência por parte dos gestores em compreender e priorizar o trabalho do técnico em saúde bucal. Sendo assim, recomenda-se que estudos futuros avaliem a variação temporal das EqSB segundo modalidades I e II.

A expansão na oferta de EqSB da ESF, encontrado em nosso estudo, também foi documentado em outros ^6^ ^,^ ^7^ ^,^ ^21^ ^,^ ^28^. Embora o incremento na oferta não seja garantia de aumento no uso de serviços de saúde ^29^ ^,^ ^30^ ^,^ ^31^, a introdução de EqSB na saúde da família e maiores taxas de cobertura destas equipes estiveram associadas a aumento no uso do sistema público ^28^ ^,^ ^32^, dada a demanda reprimida existente. Ainda, estudos apresentaram associação entre o uso de serviços da ESF por parte de mães e maior visita ao dentista entre crianças pré-escolares ^33^; além da associação entre municípios com cobertura maior que 50% de EqSB na Saúde da Família e maior uso de serviços odontológicos públicos por adolescentes ^34^. E para além do aumento, também tem sido atribuída à PNSB a diminuição das iniquidades na utilização de serviços públicos de saúde bucal ^35^.

Por fim, a evidência gerada pelo nosso estudo representa expansão contínua da oferta de EqSB da ESF nas duas primeiras décadas do século XXI. Este aumento teve um momento de impulso e outro de revés, associados, respectivamente, à implantação de política nacional de fomento à saúde bucal e à aprovação de legislação nacional de congelamento de investimentos públicos. Desigualdades observadas na tendência demonstram intencionalidade equitativa na distribuição de novos serviços com maior crescimento em municípios com maiores necessidades sociais. Tais resultados reforçam a necessidade de manutenção de investimento público contínuo e crescente, de forma a prosseguir com a ampliação da cobertura de EqSB, em resistência à ideologia neoliberal de Estado mínimo ^12^ ^,^ ^36^ e no sentido de garantir a universalidade e reduzir as iniquidades no acesso à saúde bucal no SUS ^35^ ^,^ ^37^ ^,^ ^38^ ^,^ ^39^. Sugerem-se novos estudos para melhor compreensão do impacto das políticas públicas e sociais e de fatores contextuais na variação da oferta de saúde bucal na atenção básica brasileira.

## Conclusão

Observa-se que as EqSB da ESF têm se expandido em regiões com maior necessidade social de serviços, contudo o serviço ainda é deficitário em muitas áreas necessitadas. Mais estudos são necessários a fim de investigar outros fatores que influem sobre a variação na série histórica da saúde bucal na saúde da família.
